# Delayed pharyngocutaneous fistula caused by molecular targeted therapy: a case report

**DOI:** 10.1186/s13256-022-03621-2

**Published:** 2022-10-22

**Authors:** Mioko Matsuo, Kazuki Hashimoto, Rina Jiromaru, Takashi Nakagawa

**Affiliations:** Department of Otorhinolaryngology, Graduate School of Medical Sciences, Kyushu University, 3-1-1 Maidashi, Higashi-ku, Fukuoka-City, Fukuoka Prefecture 812-8582 Japan

**Keywords:** Molecular targeted therapy, Delayed pharyngocutaneous fistula, Anastomotic leakage, Adverse events

## Abstract

**Background:**

Molecular-targeted agents used as a treatment for cancer can cause some rare and serious adverse events such as, delayed wound healing. Depending on the anticancer drug used, temporary withdrawal may be recommended before and after surgery to avoid complications. Once a surgical incision has healed and closed completely, wounds rarely open because of the initiation of molecular targeted therapy several months to years after surgery. Here, we aimed to describe a rare complication of pharyngocutaneous fistula in two patients that was thought to be caused by molecular targeted therapy.

**Case presentation:**

Case 1 involved a 64-year-old asian man who developed a delayed pharyngocutaneous fistula 3 months after total laryngectomy for laryngeal cancer. Ramucirumab, a vascular endothelial growth factor receptor inhibitor used for recurrent gastric cancer, was speculated to be involved. Case 2 involved a 71-year-old japanese man who developed a delayed pharyngocutaneous fistula 2 years and 1 month after total pharyngeal laryngectomy for pharyngeal cancer. It was speculated that imatinib, a platelet-derived growth factor receptor alpha inhibitor used for chronic myeloid leukemia, was involved.

**Conclusions:**

Although the incidence of late drug-induced anastomotic leakage is very low, when it occurs, it makes oral intake impossible for an extended period and interferes with the appropriate cancer treatment. In this report, we demonstrate the details of these two patients with such a rare complication, which may help accumulate essential data on this topic.

## Background

In recent years, cancer research has progressed significantly at the molecular level. Specifically, the development and clinical introduction of molecular-targeted agents directed at certain molecular mechanisms have been rapidly advancing. The development of molecular targeted therapy has contributed to prolonged survival of patients with several malignancies [1, 2]. In contrast, various adverse events peculiar to molecular-targeted agents, which are different from conventional cytotoxic anticancer agents, have been identified. For example, rashes and hand–foot syndrome are frequent adverse events, with an incidence of ≥ 80% characteristic of cetuximab and sorafenib, respectively [3]. In addition, although the incidence is less frequent, serious adverse events, with an incidence of approximately 1.5–2%, such as gefitinib-related interstitial lung disease [4] and trastuzumab-associated cardiac dysfunction [5], require attention and awareness.

Delayed wound healing is also a rare but severe and life-threatening adverse event caused by molecular-targeted agents [6, 7]. Normally, when tissue is damaged, various wound healing factors are released to promote cell growth and angiogenesis, thereby regenerating the damaged tissue. Vascular endothelial growth factor (VEGF) and platelet-derived growth factor (PDGF) are key factors that play important roles in the wound healing process [8, 9]. Therefore, delayed wound healing may occur if these factors are inhibited. In fact, the administration of bevacizumab, a VEGF inhibitor, induces adverse events, such as anastomotic leak and fistula formation, resulting in delayed wound healing [10, 11]. The incidence of such adverse events is low, approximately 1–2% [10]. However, because these adverse events may cause serious consequences, the causative drug should be withdrawn 6–8 weeks before and after surgery [6, 7, 11].

A few articles have reported molecular-targeted agent-induced diastasis of a surgical wound that healed and completely closed months to years after the surgery [12–14]. Here, we report two cases of delayed pharyngeal cutaneous fistula considered to be caused by molecular targeted therapy.

## Case presentation

### Case 1

**Fig. 1:**
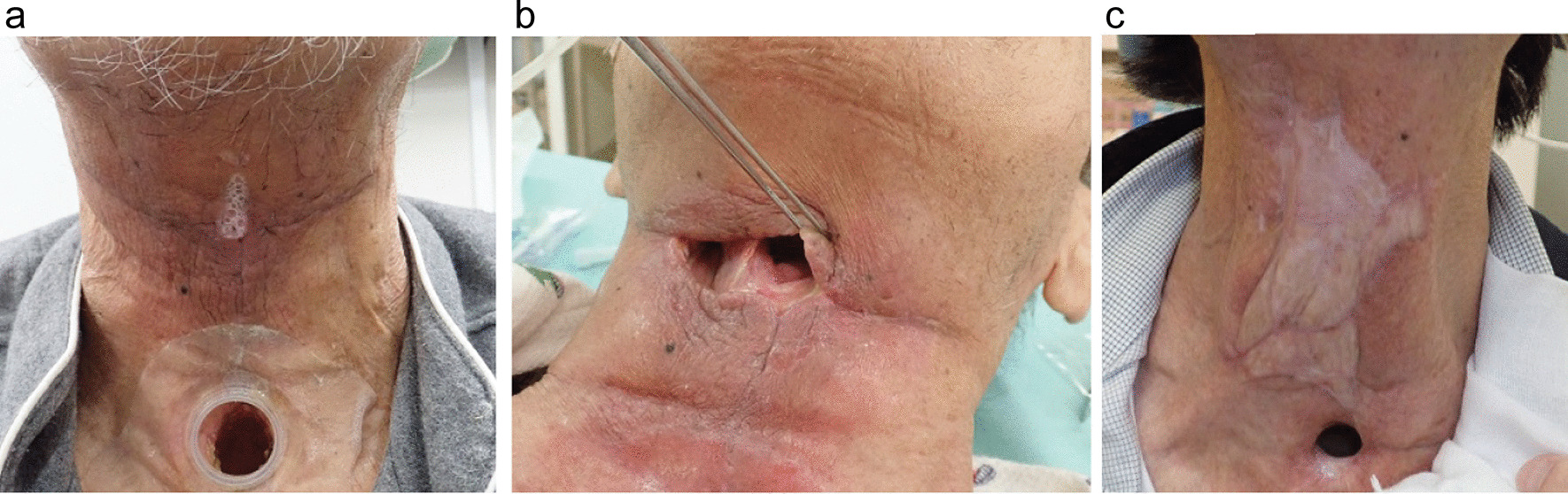
The fistula of Patient 1. **a** Pharyngocutaneous fistula (3 months after the total laryngectomy). **b** The fistula continued to increase despite discontinuing ramucirumab. **c** A complete fistula closure with two reconstructive surgeries (after 8 months from the first appearance of the fistula)

A 64-year-old asian man received cisplatin/5-fluorouracil (FU) combined with radiotherapy (60 Gy, 23 fractions) for esophageal cancer in 2014. In January 2018, he received 70 Gy (35 fractions) chemoradiotherapy with cisplatin for laryngeal cancer. In June 2018, the patient underwent total gastrectomy and postoperative adjuvant chemotherapy (capecitabine/oxaliplatin) for gastric cancer. In October 2018, total laryngectomy was performed for laryngeal cancer recurrence. There was no postoperative wound issue, oral ingestion was started 1 week after the operation, and the wound progressed uneventfully. Subsequently, gastric cancer recurred in November 2018, and combination therapy with ramucirumab and paclitaxel was initiated. In January 2019 (3 months after the previous surgery), the total laryngectomy skin incision suddenly started to leak saliva without any warning signs, such as redness, abscess formation, or skin ulceration (Fig. 1a). A delayed pharyngocutaneous fistula, which appeared 3 months after total laryngectomy, was diagnosed. Although ramucirumab was discontinued because the adverse event was thought to be caused by the drug, the fistula continued to increase in size (Fig. 1b). It was managed with various conservative treatments, for example, antibiotics, discontinuation of oral intake, enteral feeding, and negative pressure wound therapy. In February 2019 (1 month after the development of the fistula), the fistula was closed using pectoral major musculocutaneous flap reconstructive surgery. However, shortly after the operation, a new fistula appeared at a site different from the previous fistula. The new fistula was again treated with the same conservative approach. This conservative treatment was performed for approximately 7 months repeatedly for new fistulas, and a second pectoral major musculocutaneous flap reconstructive surgery was performed in August 2019 (7 months after the development of the first fistula). There was no subsequent fistula relapse, but it took 8 months from the appearance of the first fistula to complete closure (Fig. 1c). For 8 months, he was unable to orally ingest much of anything, nor could he receive chemotherapy for stomach cancer. In this patient, ramucirumab was administered for 2 months before the first surgery for recurrence of gastric cancer, and a pharyngocutaneous fistula developed 3 months after the total laryngectomy. The fistula was intractable, and complete closure was achieved after 8 months. Anemia, hypoalbuminemia, underweight, and hypothyroidism were detected in the laboratory data (Table 1). However, considering the late onset of 3 months after surgery, this case was judged to be a complication of ramucirumab, a VRGF receptor (VEGFR) inhibitor.

**Table 1:** Laboratory data at the time of fistula formation in the two cases

	Normal range	Patient 1	Patient 2
White blood cell (/μL) Hemoglobin (g/dL) Platelet (10^3^/μL) Total protein (g/dL) Albumin (g/dL) Fasting blood glucose (mg/dL) HbA1c (%) CRP (mg/dL) TSH (mIU/L) F-T4 (ng/dL) Factor XIII (%) BMI (kg/m^2^)	3300–8600 13.7–16.8 158–348 6.6–8.1 4.1–5.1 73–109 4.9–6.0 0.61–4.23 0.61–4.23 0.7–1.48 70–140 18.5–25	6630 10.0 357 6.1 3.2 75 5.1 1.8 6.57 1.02 100 16.5	7080 12.5 218 6.9 4.0 108 5.8 0.10 4.18 0.98 N.A 21.0

^*^HbA1c glycated hemoglobin, *CRP* C-reactive protein, *TSH* thyroid-stimulating hormone, *BMI* body mass index [range according to the World Health Organization (WHO) Asian-BMI classification], *N.A.* not available

### Case 2

**Fig. 2:**
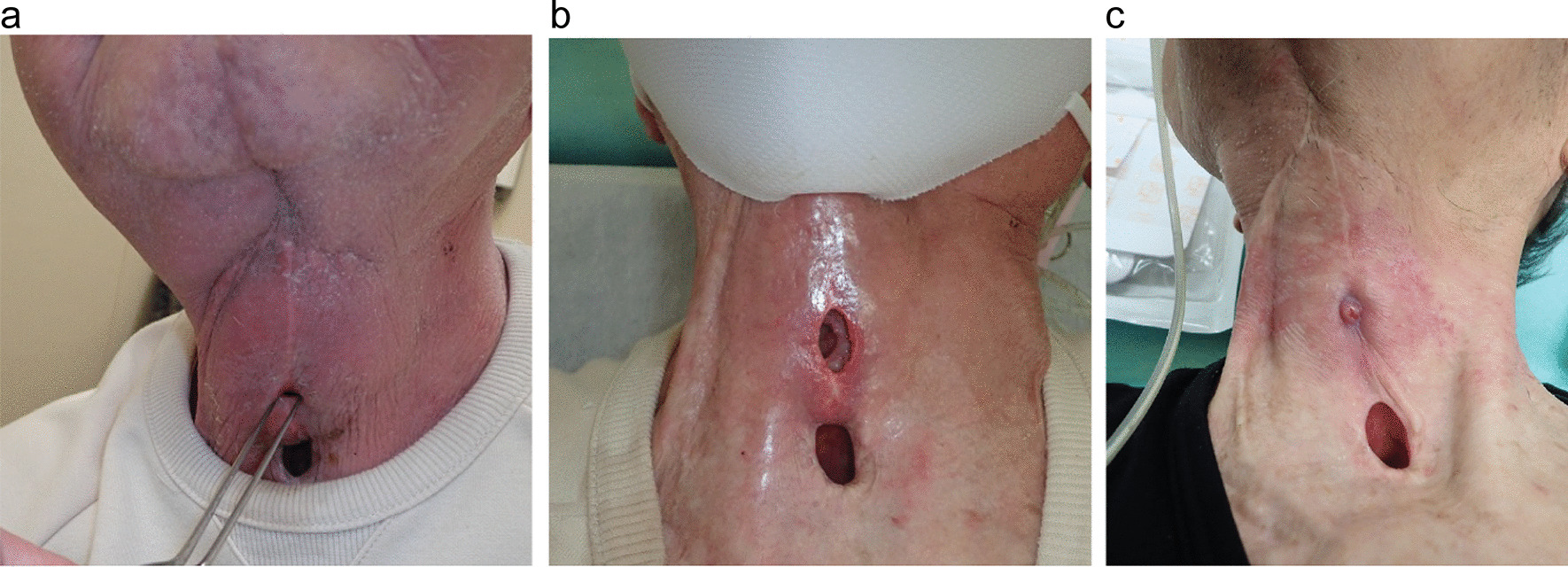
The fistula of Patient 2. **a** Pharyngocutaneous fistula (2 years and 1 month after the previous surgery). **b** The fistula continued to increase despite discontinuing imatinib. **c** Complete closure of the fistula with conservative treatment by negative pressure wound therapy (2 months from the first appearance of the fistula)

A 71-year-old japanese man underwent subtotal tongue resection, cervical dissection, and flap reconstruction for oral cancer in March 2014. In April 2019, partial pharyngeal resection, total laryngectomy, cervical dissection, and flap reconstruction were performed for laryngeal cancer. Oral ingestion was initiated 1 week after the operation, and the procedure was continued without any wound complications. He later developed chronic myelogenous leukemia, and imatinib was administered in January 2021. In May 2021 (2 years and 1 month after the previous surgery), the total laryngectomy skin incision suddenly started leaking saliva without any symptoms, such as redness, abscess formation, or skin ulceration (Fig. 2a). This was judged to be a delayed pharyngocutaneous fistula that appeared more than 2 years after total laryngectomy. Even after discontinuation of imatinib, the fistula continued to increase in size for 1 week (Fig. 2b), but it began to shrink 10 days after the drug was discontinued. The fistula closed 2 months after negative pressure wound therapy (Fig. 2c). In this patient, imatinib was administered for 2 months before surgery for chronic myelogenous leukemia. The pharyngocutaneous fistula showed delayed onset 2 years after the total laryngectomy. Mild anemia was detected in his laboratory data (Table 1). Consequently, the case was judged to be a complication of treatment with imatinib, a PDGF receptor (PDGFR) inhibitor.

## Discussion and conclusions

Pharyngocutaneous fistula is one of the major complications after laryngectomy [15–17], and the reported frequency of fistula ranges from 7.4% to 58.0% [15, 16]. However, the fistula typically occurs a median of 9–20 (range: 2–28) days after surgery [16, 18]; thus, a pharyngocutaneous fistula that develops more than several months after surgery is extremely rare. In the usual occurrence of pharyngocutaneous fistulas, which develop in approximately 9–20 days, a metaanalysis indicated that prior radiation therapy exposure [15, 17], anemia [15, 17], positive surgical margin [15, 17], supraglottic subsite or hypopharyngeal tumor site [15, 17], and chronic obstructive pulmonary disease [17] have been reported as risk factors. Meanwhile, only one case of fistula that developed a long time after the operation, similar to in our cases, has been reported to date [13]. As mentioned above, in an unusual case of delayed fistula formation, it is difficult to apply the same risk factors involved in a normal pharyngocutaneous fistula formation. Therefore, in the present cases, a strong role of VEGF and PDGF inhibitors was considered in inhibiting wound healing.

When using molecular-targeted drugs that block signaling pathways related to VEGF and PDGF, which are essential for wound healing, the risk of wound complications must be considered. Drug suspension is recommended for approximately 2 weeks to 2 months before and after surgery, depending on the half-life of each drug, to avoid the risk of wound-related complications [6]. Wound healing may be difficult to achieve after surgery in patients who have already been administered this type of molecular targeting agent. In such cases, the risk of delayed wound healing can be assumed in advance, and surgery can be planned after a safe withdrawal period, thereby reducing the incidence of wound reopening [11]. Conversely, in patients who were administered the agent after surgery, healed wounds may reopen because of drug administration.

However, as shown in the present cases, it is rare and difficult to predict the development of delayed-onset wound reopening due to a molecular-targeted drug in patients who underwent surgery months to years ago with complete closure of the surgical wound, even if the drug is associated with a risk of wound healing complications. Rare cases of delayed wound dehiscence caused by molecular-targeted drugs have been reported. Anastomotic leakage resulting from bevacizumab administration 33 months after surgery for colorectal cancer has been reported previously [12]. A pharyngocutaneous fistula was reported to have developed 23 years after administration of cabozantinib after total pharyngeal–laryngeal esophagectomy [13]. In addition, the formation of a large deep ulcer was reported after imatinib administration in a patient with skin wounds, 4 years after the wounds healed [14].

Ramucirumab, the drug used in the first case, is a monoclonal antibody that binds to VEGFR and is used to treat recurrent gastric and colon cancers. A rare incidence of adverse events (1–2%) has been reported after ramucirumab administration, including delayed wound healing and fistula formation [19, 20]. Imatinib, which is an inhibitor of PDGFR and is used for the treatment of chronic myeloid leukemia, was used in the second case. A high incidence of skin disorders (edema and rash) because of imatinib administration has been reported [21], but no reports on delayed wound healing or fistula formation incidence have been published. Imatinib has been shown to be effective in keloid scar treatment because it reduces fibroblasts and collagen formation [22]. It was speculated that imatinib use might result in delayed wound reopening because of the reduced number of fibroblasts and collagen in the wound.

Cancer cure rates have improved with the development of anticancer drugs, and the number of cancer survivors continues to increase. As a result of prolonged survival, the probability of suffering from multiple cancers in a lifetime and receiving various cancer treatments has also increased. In addition, in recent years, cancer treatment strategies have become more complicated because of the dramatically increased use of anticancer drug therapy. Adverse events associated with these treatments have also been complicated, and a new range of unexpected complications has been reported.

Anticancer drug therapy is administered along with measures against various adverse events. However, it may be possible to start anticancer drugs, such as VEGFR or PDGFR, for the treatment of new cancers different from the previous cancer treated by surgical therapy several months to years ago. In such cases, there is a concern that the drugs may be administered without evaluating the risk to the wound from the previous surgery. Although rare, anastomotic leakage makes oral intake impossible for an extended period and interferes with appropriate cancer treatment. The two cases in this report were drug-induced delayed-onset pharyngocutaneous fistulas. Detailed information on this very rare complication must be elucidated to appropriately increase awareness among healthcare professionals involved in cancer treatment.

In conclusion, drug-induced late-onset pharyngocutaneous fistula is rare. However, when it occurs, it significantly lowers the patients’ quality of life and is difficult to treat. Thus, it is indispensable to accumulate more data on such rare cases, and we believe that this report offers more insights into drug-induced late-onset pharyngocutaneous fistula.

## Data Availability

Data sharing is not applicable to this manuscript as no datasets were generated.

## Abbreviations

VEGF: Vascular endothelial growth factor
PDGF: Platelet-derived growth factor
VEGFR: VEGF receptor
PDGFR: PDGF receptor
